# Effects of Feeding Dried Fruit Pomaces as Additional Fibre-Phenolic Compound on Meat Quality, Blood Chemistry and Redox Status of Broilers

**DOI:** 10.3390/ani10111968

**Published:** 2020-10-26

**Authors:** Elena Colombino, Zenon Zduńczyk, Jan Jankowski, Luca Simone Cocolin, Achille Schiavone, Ilaria Biasato, Daniel Prieto-Botella, Elzbieta Karlińska, Monika Kosmala, Katarzyna Ognik, Maria Teresa Capucchio, Jerzy Juśkiewicz

**Affiliations:** 1 Department of Veterinary Sciences, University of Torino, Largo Paolo Braccini 2, 10095 Grugliasco, Torino, Italy; elena.colombino@unito.it (E.C.); achille.schiavone@unito.it (A.S.); mariateresa.capucchio@unito.it (M.T.C.); 2 Institute of Animal Reproduction and Food Research, Polish Academy of Sciences, 10-748 Olsztyn, Poland; z.zdunczyk@pan.olsztyn.pl; 3 Department of Poultry Science, University of Warmia and Mazury, ul. Oczapowskiego 5, 10-919 Olsztyn, Poland; janj@uwm.edu.pl; 4 Department of Agricultural, Forest and Food Sciences, University of Torino, Largo Paolo Braccini 2, 10095 Grugliasco, Torino, Italy; lucasimone.cocolin@unito.it (L.S.C.); ilaria.biasato@unito.it (I.B.); 5 Department of Surgery and Pathology, University Miguel Hernandez of Elche, Research Team on Occupational Therapy (InTeO), 03550 Alicante, Spain; daniel.prieto@goumh.umh.es; 6 Institute of Chemical Technology of Food, Lodz University of Technology, 90-924 Lodz, Poland; elzbieta.karlinska@p.lodz.pl (E.K.); monika.kosmala@p.lodz.pl (M.K.); 7 Department of Biochemistry and Toxicology, Faculty of Biology Animal Sciences and Bioeconomy, Lublin University of Life Sciences, 20-950 Lublin, Poland; kasiaognik@poczta.fm

**Keywords:** broiler, blood lipids, dried fruit pomace, phenolic compounds, tissue redox status

## Abstract

**Simple Summary:**

Fruit juice production resulted in a considerable amount of by-products that are rich in phenolic compounds. Several studies have already reported that polyphenols seemed to have antioxidant, anti-inflammatory and hypolipidemic properties. For this reason, fruit extracts have been widely used as a human food supplement for health promotion and disease prevention. However, little information about their application in animal feeds is available. The aim of this study was to investigate whether 3% or 6% apple, blackcurrant and strawberry dietary inclusion could have a positive effect on meat quality, blood chemistry and redox status of broiler chickens. Overall, the obtained results seem encouraging as both 3% and 6% fruit pomaces diets did not impair carcass traits and meat quality. Moreover, fruit pomaces groups showed lower blood triglycerides and improved renal function with lower creatinine level. Regarding antioxidant activity, all fruit pomaces improved the redox status in liver, breast and blood. No differences have been recorded between 3% and 6% diets. From a productive and biological point of view, the use of fruit pomaces in broiler chicken nutrition seems to be promising, in particular, 3% dietary inclusion seems to be preferable as higher fibre level can impair nutrient digestibility in poultry.

**Abstract:**

The present study investigated the effects of apple (A), blackcurrant (B) and strawberry (S) dried pomaces on meat quality, blood chemistry and redox status of broiler chickens. A total of 480 Ross-308 male broilers were divided into 8 dietary treatments containing 3% and 6% of cellulose preparation (C), A, B or S. Six birds/group were slaughtered at 35 days of age and blood samples were collected. Carcass traits and meat quality were determined on the Pectoralis major muscles, recording nonsignificant differences. Antioxidant activity was evaluated in serum, liver and breast muscle. In serum, fruit pomaces lowered triglycerides, creatinine and atherogenic index (*p* < 0.05). Regarding redox status, in serum, ACW (antioxidant capacity of water-soluble substances) and ACL (antioxidant capacity of lipid-soluble substances) were greater in A (*p* < 0.001). In breast, ACW and ACL were higher in B and S compared to C (*p* < 0.05). In liver, ACL was greater in B and S compared to C (*p* < 0.001) and in higher dosage compared to low (*p* = 0.036). GSSG (oxidized glutathione) concentration was lower in A, whereas A, B and S presented a higher GSH (reduced glutathione)/GSSG ratio. The results showed that fruit pomaces could represent promising feed ingredients for broilers, improving serum, meat and tissue antioxidant parameters.

## 1. Introduction

Jams and juices production generate a large amount of by-product consisting in fruit pomaces with or without seeds that can be an important and inexpensive dietary additive. The use of these pomaces in animal feed has been widely investigated with the aim of obtaining foods with high nutritional value and beneficial effects on consumer health [1,2]. In fact, it is well known that fruit pomaces, such as apple, blackcurrant or strawberry can be a very valuable source of natural phenolic compounds with antioxidant, anti-inflammatory and hypolipidemic properties, such as flavan-3-ols, anthocyanins, ellagitannins and phenolic acids [3]. Additionally, the content of beta-carotene, vitamins, trace elements, and polyunsaturated fatty acids (PUFAs) in pomaces is noteworthy [4]. Previous works had already demonstrated that their use in laboratory and farm animals is able to reduce hypercholesterolaemia and hypertriglyceridemia [5] as well as to efficiently support the maintenance of a correct redox status [6,7]. The enhancement of the antioxidant activity through natural feed ingredients has received major attention in the last decades research. In fact, in the body, endogenous and exogenous free radical formation occurs physiologically and the exposure to environmental oxidants cannot be avoided [8]. As a consequence, if the redox status is not maintained, an imbalance between production and destruction of reactive oxygen species (ROS) occurred producing oxidative stress. Fruit pomaces seemed to be a promising natural source of antioxidant compounds as reported by Jankowski et al. [9]. In their study, they observed beneficial effects on blood antioxidant parameters in turkeys fed diets supplemented with apple, strawberry or blackcurrant pomace in terms of increased vitamin C concentration, integral antioxidant capacity of lipophilic and hydrophilic substances and decreased lipid peroxide levels.

Moreover, phenolic compounds can affect meat quality by increasing resistance to oxidizing agents, enhancing the integrity of cell membranes, inhibiting water loss from cells, improving colour stability and sensory properties such as taste and aroma and improving its eating quality [10,11]. It also appears that the use of dried fruit pomaces in animal feeding may enrich animal products, such as meat, with substances beneficial for human health: vitamins, unsaturated fatty acids (UFA), minerals and antioxidants [11]. Taking this into consideration, it can be stated that the presence of natural antioxidants compounds in commercially available products might influence the consumers’ choice of products [12]. Nowadays consumers are very sensible concerning these information and they are showing greater interest in foods that contain bioactive or functional components which will give additional benefits to their health status [13].

However, inclusion of fruit pomaces to poultry diet is not free of risks as they are rich in fibre and prone to decreased growth performances when included at excessive doses in diet [14]. It is also to state that the use of fruit pomaces in animal nutrition could be made difficult by the seasonality of fruit production. Considering this background, the present study aimed to investigate whether an incremental dietary incorporation of highly fibrous dried fruit pomace into animal feed could produce some beneficial effects on meat quality and redox balance status of tissues, liver and blood in broilers.

## 2. Materials and Methods

### 2.1. Birds, Management and Diets

The experiment was carried out at the Research Laboratory of the Department of Poultry Science, University of Warmia and Mazury in Olsztyn (Poland). A total of 480 Ross 308 male broilers at 1-day of age (Animex Group, Sokolka, Poland) were randomly allotted to 8 dietary treatments (6 replicates/treatment; 10 birds/replicate). The experimental protocol was approved by the Local Animal Care and Use Committee (Decision No. 2/2018; Olsztyn, Poland), and the study was carried out in accordance with EU Directive 2010/63/EU for animal experiments.

Apple (A), blackcurrant (B) and strawberry (S) dried pomaces (Agro-Bio-Produkt Sp. z.o.o., Grodkowice, Poland) were added to the experimental diets at 3% (L, low cellulose) or 6% inclusion rate (H, high cellulose). The fruit pomaces were dried in the SB-1.5 rotary drum dryer (AGROMECH Co., Rogozno Wlkp., Poland) for biomass residues. A control diet (C) was formulated and VITACEL® cellulose (Rettenmaier, Warsaw, Poland) preparation was added as fibre component (L and H, respectively). Each diet was formulated for two different feeding phases: starter (days 1–14) and finisher (day 15–35) (Table 1). The nutritional value of the experimental diets was consistent with broilers nutrient requirements [15].

### 2.2. Chemical Analysis of Fruit Pomaces and Experimental Diets

Experimental diets and fruit pomaces were analysed to establish dry matter, crude protein, crude fat, crude fibre, crude ash, total dietary fibre (TDF) and soluble and insoluble fibre fraction (SDF and IDF, respectively) according to AOAC International [16]. High-Performance Liquid Chromatography Diode Array Detector (HPLC DAD, Smartline chromatograph–Knauer, Berlin, Germany) as then used to determine the polyphenols content in fruit pomaces and diets as reported by Colombino et al. [17] (Table 1 and Table 2). The dried fruit pomaces differed in the content and composition of nutrients, nonnutrients, fibre and polyphenolic fraction (Table 2). Regarding fibre content, TDF and SDF were similar in A (60.8% and 9.1%) and B pomaces (60.6% and 7.7%) and lower in S pomace (52.8% and 0.40%). IDF content was similar in all the pomaces (51.7–52.9%). Considering polyphenols content, procyanidins were the most abundant polyphenols in all the pomaces. In particular, A pomace showed the lowest polyphenols concentration (8.43 mg/g), composed by procyanidins (6.75 mg/g), quercetin glucosides (0.83 mg/g), phlorizin (0.55 mg/g) and chlorogenic acid (0.26 mg/g). The polyphenols were more than 3-fold higher in B pomace than in A pomace (26.7 mg/g) containing procyanidins (22.5 mg/g), anthocyanins (3.74 mg/g) and myricetin glycosides (0.34 mg/g). S pomace showed the highest concentration of polyphenols (28.9 mg/g) composed by procyanidins (15.8 mg/g), ellagitannins (11.2 mg/g) and small content of tiliroside, ellagic acid, quercetin glycosides and anthocyanins (0.85, 0.57, 0.27, and 0.14 mg/g, respectively).

### 2.3. Slaughtering Procedures

The trial lasted 35 days. At day 35, 6 birds/treatment (1 bird/replicate) were selected on the basis of pen average live weight, tagged and fasted for 8 h. The birds were electrically stunned (400 mA, 350 Hz), hung on a shackle line and exsanguinated by a unilateral neck cut. After a 3 min bleeding period, the birds were scalded at 61 °C for 60 s, defeathered in a rotary drum picker for 25 s, and manually eviscerated (nonedible viscera: full crop, proventriculus, small intestine and caeca).

### 2.4. Carcass Traits and Meat Quality Parameters

Weights of breast, thigh, shank, gizzard, liver, heart and abdominal fat were immediately recorded after slaughtering and expressed as relative weight of the carcass (%). Samples of chicken left breast muscle (2.5 cm × 2.5 cm × 2.5 cm) were freeze-dried, milled to a fine powder and stored at −80 °C until further analysis.

After 24 h of chilling, meat quality parameters (pH, colour, fat and lean ratio) were determined on the Pectoralis major muscles. To prevent muscle surface drying after slaughtering, breast muscles were individually stored in plastic bags at 4 °C until meat quality analysis. The pH of breast muscles was measured using a Testo Ltd. 206-pH2 meter (Testo Ltd, Pruszkow, Poland). The Hunter L* (lightness, a lower value indicates a darker colour), a* (redness, a higher positive value indicates a higher contribution of redness) and b* (yellowness, a higher value indicates a higher contribution of yellowness) values were determined on the medial surface of each right breast muscle immediately after removal using a MiniScan XE Plus colour difference meter (Hunter Associates Laboratory, Inc., Reston, VA, USA). Three measurements for each parameters/animal were performed and the average value was recorded. The fat and lean/fluid mass of breast muscle was determined shortly after dissection by time-domain nuclear magnetic resonance using the minispec LF 90II analyser (Bruker, Karlsruhe, Germany).

### 2.5. Serum Biochemical Parameters

Blood samples were collected at slaughtering, before stunning from the wing vein of 36 birds (6 birds/diet). An aliquot of 2.5 mL was placed into heparin tubes. Subsequently, the tubes were centrifuged at 380 g for 10 min and the obtained serum was stored at −80 °C. The alanine-aminotransferase (ALT), aspartate-aminotransferase (AST), gamma glutamyl transferase (GGT), alkaline phosphatase (ALP), triglycerides, HDL (high-density lipoprotein)/non-HDL-cholesterol, creatinine, uric acid, Ca, P and Mg concentrations in serum were estimated using a biochemical analyser (Pentra C200, HORIBA, Tokyo, Japan). The atherogenic index of plasma (AIP) was calculated as follows: log(triglycerides/HDL) as reported by Fernández-Macías et al. [18].

### 2.6. Antioxidant Parameters

Indicators of antioxidant activity were evaluated in breast muscle, liver and serum. Photochem® apparatus (Analytik Jena, Leipzig, Germany) was used to evaluate the antioxidant capacity of water-soluble substances (ACW) and the antioxidant capacity of lipid-soluble substances (ACL) using a photoluminescent (PCL) method based on the scavenging activity against the superoxide anion radical [19]. PCL ACW and PCL ACL kits were obtained from Analytik Jena Ag (Jena, Germany).

Ferric reducing antioxidant potential (FRAP) assay was carried out according to microplate method described by Horszwald and Andlauer, [12] and the results were expressed as milligram of Trolox per gram of dry matter of breast/liver or milligram of Trolox per millilitre of serum.

Additionally, thiobarbituric acid reactive substances (TBARS) levels were determined in liver for measuring lipid peroxidation according to the method described by Ognik and Wertelecki, [20]. Results were expressed in micromole of malondialdehyde (MDA) per kilogram of tissue. The reduced glutathione (GSH) and oxidized glutathione (GSSG) concentrations were also determined in liver by an enzymatic recycling method described by Rahman et al. [21].

### 2.7. Histopathological Investigations

The slaughtered birds (6/diet) were submitted to anatomopathological examination. Liver, spleen, thymus and bursa of Fabricius were collected and fixed in 10% buffered formalin, embedded in paraffin wax block, sectioned at a thickness of 5 μm, mounted on glass slides and stained with haematoxylin and eosin. The observed histopathological alterations were evaluated using a semiquantitative scoring system (absent—0, mild—1, moderate—2, and severe—3). All the slides were blindly evaluated by two different pathologists.

### 2.8. Statistical Analysis

Individual birds were considered as experimental units to analyse meat quality, blood and tissue antioxidants parameters. R software (version 4.0.2, R Foundation for Statistical Computing; Vienna, Austria) was used to perform the Shapiro–Wilk test in order to check the normality of the data distribution before statistical analyses. Data were described by mean and standard deviation (SD). Bivariate analysis was performed by Kruskal–Wallis and one-way ANOVA tests. Data were also analysed by a robust two-way ANOVA test (method of trimmed means) in order to evaluate separately the effect of diet (A, B and S), dosage (H or L) and interaction between them. The interactions between the levels of the fixed factors were evaluated by robust pairwise comparisons. Two-way ANOVA results were discussed only when a significant difference was found among the treatment groups by Kruskal–Wallis or one-way ANOVA test. *p* values ≤ 0.05 were considered statistically significant.

## 3. Results

### 3.1. Carcass Traits and Meat Quality Parameters

The applied dietary treatments did not affect either the carcass traits or the meat quality parameters (Table 3). However, a slightly significant difference regarding fat tissue content and lean tissue and fluid content was recorded among the treatment groups (*p* = 0.068). In fact, these two parameters were influenced by fruit pomaces (*p* = 0.008): A, B and S diets increased fat tissue content and decreased lean tissue and fluid content of meat compared to control diet.

### 3.2. Serum Biochemical Parameters

Data regarding serum biochemical parameters are reported in Table 4.

Considering serum lipids, triglycerides showed significant differences among the treatment groups (*p* < 0.001), depending on fruit pomaces (*p* < 0.001). In fact, A, B and S groups showed lower serum triglycerides when compared to control group. Moreover, also AIP significantly differed among the groups, being greater in control groups than in fruit pomace diets (*p* < 0.001).

Regarding renal function, creatinine levels were significantly lower in BH, BL, SH and SL groups compared to control (*p* = 0.05). The two-way ANOVA showed that creatinine levels were influenced by fruit pomaces (*p* = 0.021): chickens fed with B and S diets showed lower serum creatinine compared to C groups.

Finally, ALT showed a slightly significant difference among groups (*p* = 0.061), due to fruit pomace (*p* = 0.021). In fact, A and B diets presented a lower AST activity than C group.

### 3.3. Antioxidant Parameters

Serum, breast and liver antioxidant parameters are summarized in Table 4 and Table 5.

In serum, ACW and ACL showed significant differences among the groups (*p* < 0.001). In particular, ACW depended on pomace (*p* = 0.008) and dosage (*p* = 0.004), being greater in A pomace than in C, B and S groups and in H diets than in L diets. In addition, ALC depended on pomace (*p* = 0.003) and interaction between pomace and dosage (*p* = 0.027), being lower in B and S groups.

In breast, ACW and ACL significantly differed among treatments (*p* = 0.019 and *p* = 0.029, respectively), depending both on pomace (*p* = 0.007 and *p* = 0.008). In fact, ACW was higher in B and S diets compared to C, whereas ACL increased in S diet compared to the other treatments.

In liver, ACL showed significant differences among treatments (*p* < 0.001), depending on pomace (*p* = 0.001), dosage (*p* = 0.036) and the interaction between them (*p* = 0.043). Particularly, ACL was greater in B and S groups compared to C and in H dosage compared to L. In addition, GSSG and GSH/GSSG were significantly different among the experimental groups (*p* = 0.003 and *p* < 0.001, respectively), depending both on pomace (*p* = 0.052 and *p* = 0.017) and interaction between pomace and dosage. All the fruit pomaces groups presented a higher GSH/GSSG ratio than C, while GSSG concentration was lower in A group than in the other diets.

### 3.4. Histopathological Investigations

Histopathological alterations were identified in all the organs for all the dietary treatments. Spleen showed mild white pulp hyperplasia and bursa of Fabricius showed from mild to moderate follicular depletion. Mild to severe vacuolar degeneration and mild lymphoid tissue activation was observed in liver. No alterations were observed in thymus. No significant differences were observed among the dietary treatments on the evaluated parameters (*p* > 0.05) (Table 6).

## 4. Discussion

Fruit pomaces have been shown to have beneficial antioxidant activity and they can enrich animal products with health-promoting benefits for consumers [9,13]. The current study evaluated whether fruit pomaces diets can have beneficial effects on meat quality, serum parameters and redox status of broilers.

In the present experiment, dietary crude fibre content was levelled out at 3.2–3.3% and 3.9–4.0% in L and H treatments, respectively. This is in accordance with the requirements for crude fibre in broiler [22] as higher fibre concentration may cause negative consequences on growth performances, reducing protein and fat digestibility [23]. Regarding polyphenols content, A pomace showed the lowest content, whereas it was more than 3-fold higher in B and S pomaces. Each pomace also differs in terms of major phenolic compounds amounts. In fact, A pomace was richer in procyanidins, B pomace in procyanidins and anthocyanins, whereas in S pomace, procyanidins and ellagitannins were the dominant fractions. This is in accordance with previous works investigating the phenolic compounds content in fruit pomaces [24,25].

There is no doubt that dietary polyphenols showed anti-ROS activity but also negative effects on nutrient digestibility, when provided at relatively high amounts, have been described [26]. Nyamambi et al. [27] reported that dietary condensed polyphenols may easily diminish the activity of small intestinal mucosal enzymes, thus depress the digestion rate of dietary protein and carbohydrates. A significant decrease in intestinal protein digestibility was reported also in broilers fed diets containing more than 2.5 mg/g of grape polyphenols extract [28]. In the present experiment, the highest total polyphenol concentration was noted in the starter period BH diet (1.37 mg/g) but it did not exceed the 2.5 mg/g of diet.

In the present study, no effect on carcass traits and meat quality was observed. Similar findings were reported by Juskiewicz et al. [29] in fruit pomaces-fed turkeys. On the contrary, Mazur-Kuśnirek et al. [30] reported an higher content of breast muscle on the carcass and a lower contribution of yellowness (b*) in the breast muscles of broilers fed with polyphenols-enriched diets. Jiang et al. [31] also found an increased pH and an increased L* of meat colour in broiler fed with soybean isoflavone rich in polyphenols. However, A, B and S diets slightly increased fat tissue and decreased lean tissue and fluid content of meat compared to control diet. This slightly increase in the fat tissue percentage should be regarded as nutritionally advisable as the intramuscular fat positively affect flavour perception, generation and stability [32].

The available literature showed that dietary plant polyphenols added as fibre-bound compounds or purified extracts could be considered as potent antioxidant molecules in animal nutrition [4]. However, there is no doubt that the application of dietary polyphenols must be judicious as pro-oxidative action of low-molecular-weight compounds has also been reported [33]. In the present study, the dietary application of three fruit pomaces was accompanied by health-promoting changes of different antioxidant mechanisms in serum, breast and liver. In serum, ACW depended on pomace (*p* = 0.008) and dosage (*p* = 0.004), being greater in A pomace and in H diets. In addition, ALC depended on pomace (*p* = 0.003) being greater in A and C groups. In breast, ACW and ACL were influenced only by pomace (*p* = 0.007 and *p* = 0.008). In fact, ACW was higher in B and S diets, whereas ACL increased in S diet compared to the other treatments.

In liver, ACL depended on pomace (*p* = 0.001), dosage (*p* = 0.036) and the interaction between them (*p* = 0.043), being greater in B and S groups and in H dosage compared to L. Regarding GSSG and GSH/GSSG, all the fruit pomaces groups presented a higher GSH/GSSG ratio than C while GSSG concentration was lower in A group than in the other diets. This is in accordance with Jankowski et al. [9] that observed an increased serum ACL and ACW in turkeys fed A and S diets. On the contrary, Vossen et al. [34] showed no significant influence on serum antioxidant status in broiler chickens receiving dietary antioxidant supplementation from grape seed or tomato. Moreover, Leskovec et al. [35] found no differences in ACW activity in serum and breast in chickens fed with a linseed oil-enriched diets, whereas they recorded an increase in ACL breast activity. The heterogeneity of these results presented in literature can be due to the fact that different known and unknown synergistic effects of fat- and water-soluble antioxidants could indirectly affect the oxidative status when supplements containing multiple antioxidants are provided to animals [34].

Considering the overall antioxidant activity, it is interesting to note that fruit pomaces generally increased ACW and/or ACL activity in serum, meat and liver, i.e., apple diet showed the greatest effect in serum and liver, whereas blackcurrant and strawberry diets showed the greatest effects in meat and liver. Regarding cellulose level, it showed little influence on antioxidant parameters in serum and liver, while it seemed to not modify meat ACW and ACL. As the cellulose levels seemed to have little effects on serum and tissue antioxidant parameters, L cellulose should be preferable considering that high levels could impair nutrient digestion [23].

All the aforementioned changes in serum and tissues antioxidant activity should be attributed, at least partially, to phenolic metabolites that are able to infiltrate hydrophobic areas (such as the lipid bilayers of cells) and hydrophilic areas (e.g., blood serum) [24,25]. As the hydrophobic properties vary among the different phenolic compounds [36], differences in the relative abundance of each polyphenol might be the reason for variations in the antioxidant effects of A, B and S diets. Serum biochemical parameters were also positively modulated by fruit pomaces. In particular, triglycerides and AIP depended on fruit pomaces (*p* < 0.001), being lower in A, B and S groups compared to C. This is a positive finding and it is in accordance with literature, as Meydani and Hasan [37] reported that anthocyanins extracted from blueberry could lower serum triglycerides and leptin level, an hormone that reduces triglycerides formation in various organs by increasing free fatty acids oxidation and decreasing its esterification. It has been also suggested that fruit pomaces are rich sources of PUFAs, like linoleic acid and α-linolenic acids, which may additionally contribute to the lipid-lowering activity of the pomace diets observed in the present experiment [1,29]. Fruit pomaces, particularly B and S diets also reduced blood creatinine level compared to C (*p*= 0.021). Even if all groups showed normal creatinine level, an increase in dietary fibre intake has been reported to reduce blood creatinine levels in humans [38]. It could be hypothesize that also in poultry, higher level of dietary fibre could increase creatinine degradation by bacterial creatinase throughout the bowel and thus potential loss to the creatinine pool [38].

Finally, fruit pomaces diets did not significantly influence either the development or the severity of the histopathological alterations detected in the broiler chickens of the current research. These results seem to suggest that the applied fruit pomaces dietary inclusion showed no negative effect on animal health.

## 5. Conclusions

In conclusion, dietary application of apple, blackcurrant and strawberry pomaces could strengthen serum and tissue antioxidant activity along with improving the serum lipid profile in broilers with no negative effects on meat quality and carcass traits. Cellulose level did not influence antioxidant parameters in meat, suggesting that lower level of cellulose should be preferred in poultry diet in order to avoid negative effects on nutrient digestibility. However, the present study had several limitations concerning low statistical power due to the small sample size. Further studies with a higher number of animals seem necessary to better understand which doses and which fruit pomaces should be more suitable for poultry nutrition.

## Figures and Tables

**Table 1:** Ingredients (%) and nutritional values of the experimental diets.

	Starter Diets (Days 1–14)								Grower Diets (Days 15–35)							
	CL	CH	AL	AH	BL	BH	SL	SH	CL	CH	AL	AH	BL	BH	SL	SH
Wheat	43.0	43.0	37.9	32.9	39.2	35.5	39.3	35.5	47.0	46.9	42.0	36.9	43.3	39.6	43.3	39.6
Corn	20.0	20.0	20.0	20.0	20.0	20.0	20.0	20.0	20.0	20.0	20.0	20.0	20.0	20.0	20.0	20.0
Soybean meal	29.6	29.6	30.5	31.3	29.5	29.4	29.5	29.3	24.5	24.5	25.3	26.1	24.4	24.3	24.3	24.1
Cellulose	0.70	1.38	–	–	–	–	–	–	0.70	1.38	–	–	–	–	–	–
Apple pomace	–	–	3.00	6.00	–	–	–	–	–	–	3.00	6.00	–	–	–	–
Blackcurrant pomace	–	–	–	–	3.00	6.00	–	–	–	–	–	–	3.00	6.00	–	–
Strawberry pomace	–	–	–	–	–	–	3.00	6.00	–	––	–	–	–	–	3.00	6.00
Soybean oil	2.83	2.83	4.02	5.22	3.58	4.33	3.62	4,42	4.47	4.52	5.67	6.86	5.23	5.98	5.27	6.07
Sodium bicarbonate	0.15	0.15	0.15	0.15	0.15	0.15	0.15	0.15	0.15	0.15	0.15	0.15	0.15	0.15	0.15	0.15
Fodder salt	0.23	0.23	0.23	0.23	0.23	0.23	0.23	0.23	0.23	0.23	0.23	0.23	0.23	0.23	0.23	0.23
Limestone	1.29	1.29	1.27	1.25	1.28	1.27	1.28	1.27	1.09	1.02	1.08	1.06	1.08	1.07	1.09	1.08
Mon-Ca phosphate	0.87	1.19	1.60	1.64	1.60	1.64	1.60	1.64	0.53	0.53	1.26	1.29	1.26	1.29	1.26	1.29
DL-Methionine 99	0.37	0.37	0.38	0.39	0.39	0.41	0.39	0.41	0.34	0.34	0.35	0.36	0.36	0.38	0.36	0.38
L-Lysine 99	0.33	0.33	0.33	0.32	0.35	0.38	0.36	0.38	0.35	0.35	0.34	0.33	0.37	0.39	0.37	0.39
L-Threonine	0.15	0.15	0.15	0.15	0.16	0.18	0.16	0.18	0.16	0.18	0.16	0.16	0.17	0.19	0.17	0.19
Vitamin premix ^1^	0.50	0.50	0.50	0.50	0.50	0.50	0.50	0.50	0.50	0.50	0.50	0.50	0.50	0.50	0.50	0.50
Nutrient composition																
Dry matter	87.7	87.0	85.9	83.5	85.9	83.4	85.9	83.4	87.8	87.1	86.0	83.6	86.0	83.4	86.0	83.5
Crude protein, %DM	21.5	21.5	21.5	21.5	21.5	21.5	21.5	21.5	19.5	19.5	19.5	19.5	19.5	19.5	19.5	19.5
ME, kcal	2950	2950	2950	2950	2950	2950	2950	2950	3100	3100	3100	3100	3100	3100	3100	3100
Crude fat, %DM	4.80	4.80	5.97	7.17	5.89	6.97	5.83	6.86	6.46	6.50	7.63	8.81	7.55	8.64	7.49	8.52
Crude fibre, %DM	3.33	3.98	3.23	3.98	3.32	3.98	3.32	3.97	3.23	3.88	3.23	3.88	3.22	3.88	3.22	3.87
Lysine, %DM	1.34	1.34	1.34	1.34	1.34	1.34	1.34	1.34	1.18	1.18	1.18	1.18	1.18	1.18	1.18	1.18
Methionine, %DM	0.73	0.73	0.73	0.73	0.73	0.73	0.73	0.73	0.62	0.62	0.62	0.62	0.62	0.62	0.62	0.62
Met + Cys, %DM	1.11	1.11	1.11	1.11	1.11	1.11	1.11	1.11	0.97	0.97	0.97	0.97	0.97	0.97	0.97	0.97
Threonine, %DM	0.92	0.92	0.92	0.92	0.92	0.92	0.92	0.92	0.83	0.83	0.83	0.83	0.83	0.83	0.83	0.83
Ca, %DM	1.01	1.01	1.01	1.01	1.01	1.01	1.01	1.01	0.85	0.85	0.85	0.85	0.85	0.85	0.85	0.85
P available, %DM	0.38	0.38	0.38	0.38	0.38	0.38	0.38	0.38	0.42	0.42	0.42	0.42	0.42	0.42	0.42	0.42
Na, %DM	0.17	0.17	0.17	0.17	0.17	0.17	0.17	0.17	0.15	0.15	0.15	0.15	0.15	0.15	0.15	0.15
Fibre fraction ^2^																
TDF	14.8	15.0	14.4	14.9	12.8	13.8	12.8	15.1	12.8	15.3	17.7	17.5	13.1	15.1	13.8	14.4
IDF	12.7	13.3	13.3	13.8	12.3	13.0	12.2	13.2	12.4	14.3	14.7	15.4	11.8	13.1	13.0	13.9
SDF	2.00	1.70	1.00	1.10	0.60	0.70	0.60	1.90	0.40	1.00	3.00	2.10	1.20	2.00	0.70	0.50
Polyphenols (mg/g)^2^																
Total ^3^	0.91	0.98	0.95	1.06	0.97	1.37	1.00	1.27	0.81	0.88	0.83	1.00	0.84	1.24	0.91	1.15
Flavan-3-ols	0.18	0.18	0.27	0.41	0.86	1.27	0.61	1.05	0.15	0.17	0.35	0.47	0.61	1.09	0.45	0.94
Procyanidins	0.18	0.18	0.27	0.41	0.86	1.27	0.61	1.05	0.14	0.17	0.34	0.47	0.61	1.09	0.44	0.94

CL: control diet low dosage; CH: control diet high dosage; AL: 3% inclusion level of apple pomace; AH: 6% inclusion level of apple pomace; BL: 3% inclusion level of blackcurrant pomace; BH: 6% inclusion level of blackcurrant pomace; SL: 3% inclusion level of strawberry pomace; SH: 6% inclusion level of strawberry pomace; ME: metabolizable energy; Mon-Ca phosphate: monocalcium phosphate; TDF: total dietary fibre; ME: metabolizable energy; IDF: insoluble dietary fibre; SDF: soluble dietary fibre. ^1^ Contents per kilogram feed: vitamin A, 12500 IU; vitamin D3, 3500 IU; vitamin E, 50 mg; vitamin K3, 3 mg; vitamin B1, 3 mg; vitamin B2, 8 mg; vitamin B6, 5 mg; vitamin B12, 0.025 mg; biotin, 0.25 mg; calcium pantothenate, 12 mg; nicotinic acid, 50 mg; folic acid, 2 mg; choline chloride, 400 mg; Fe, 50 mg; Mn, 100 mg; Zn, 100 mg; Cu, 12 mg; I, 1 mg; Se, 0.3 mg. ^2^ Analysed content; ^3^ Calculated on milligram of gallic acid equivalents per gram of diet; according to Folin–Ciocalteu method.

**Table 2:** Nutrient composition and polyphenols content of the fruit pomaces.

	Apple Pomace	Blackcurrant Pomace	Strawberry Pomace
Dry matter (%)	92.4	93.7	93.2
Crude ash (%)	1.10	3.89	8.01
Crude protein (%)	6.64	15.5	16.4
Crude fat (%)	2.63	13.8	10.4
Crude fibre (%)	22.0	19.8	31.4
Macroelements:			
Ca	0.09	0.36	0.37
K	0.23	0.39	0.15
P	0.16	0.33	0.43
Mg	0.04	0.17	0.10
Na	<0.01	<0.01	<0.01
Fibre (%)			
Total dietary fibre	60.8	60.6	52.8
Insoluble dietary fibre	51.7	52.9	52.5
Soluble dietary fibre	9.10	7.70	0.40
Polyphenols (mg/g)			
Total polyphenols	8.43	26.7	28.9
Anthocyanins	0.00	3.74	0.14
Chlorogenic acid	0.26	0.00	0.00
Ellagic acid	0.00	0.00	0.57
Ellagitannins	0.00	0.00	11.2
Agrimoniin	0.00	0.00	4.19
Myricetin glycosides	0.00	0.34	0.00
Kaempferol glycosides	0.00	0.00	0.04
Kaempferol	0.00	0.03	0.06
Quercetin glycosides	0.83	0.00	0.27
Quercetin	0.03	0.06	0.00
Phloridzin	0.55	0.00	0.00
Tiliroside	0.00	0.00	0.85
Flavan-3-ols	6.76	22.5	15.8
Procyanidins	6.75	22.5	15.8
Free catechins	0.02	0.01	0.03

**Table 3:** Carcass traits and meat quality parameters.

	Groups								*p*-Value *	Pomace (P)				Dosage (D)		*p*-Value P ^#^	*p*-Value D ^#^	*p*-Value P × D ^#^
	CH	CL	AH	AL	BH	BL	SH	SL		C	A	B	S	L	H			
pH 24 h	5.74 (0.2)	5.64 (0.1)	5.84 (0.1)	5.78 (0.1)	5.79 (0.1)	5.84 (0.1)	5.75 (0.1)	5.78 (0.2)	0.314	5.69 (0.1)	5.81 (0.1)	5.82 (0.1)	5.77 (0.1)	5.78 (0.1)	5.77 (0.1)	0.016	0.909	0.534
Colour																		
L *	55.11 (3.6)	55.56 (2.9)	56.16 (3.0)	56.91 (6.9)	56.07 (3.1)	55.84 (1.9)	55.81 (2.9)	58.11 (4.9)	0.934	55.3 (3.1)	56.5 (5.1)	56.0 (2.5)	57.0 (4.0)	55.8 (3.5)	56.6 (3.8)	0.824	0.579	0.717
a *	6.12 (1.8)	6.24 (1.04)	4.87 (1.08)	5.16 (2.3)	6.02 (1.5)	5.33 (1.8)	5.45 (1.38)	5.20 (0.6)	0.700	6.19 (1.4)	5.02 (1.7)	5.68 (1.6)	5.33 (1.0)	5.62 (1.5)	5.49 (1.4)	0.667	0.961	0.864
b *	16.76 (2.4)	16.58 (1.4)	16.64 (3.0)	14.63 (0.7)	16.49 (2.1)	15.21 (1.9)	16.27 (1.2)	15.76 (2.3)	0.517	16.7 (1.8)	15.6 (2.3)	15.9 (2.0)	16.0 (1.7)	16.5 (1.9)	15.5 (1.9)	0.390	0.065	0.887
Fat tissue (%)	15.39 (1.3)	15.88 (2.1)	16.88 (1.6)	17.10 (1.4)	17.17 (0.6)	16.45 (1.5)	18.03 (1.2)	17.66 (1.8)	0.068	15.6 ^a^ (1.6)	17.00 ^b^ (1.4)	16.8 ^b^ (1.1)	17.8 ^b^ (1.5)	16.9 (1.5)	16.8 (1.5)	0.008	0.346	0.731
L&F (%)	84.61 (1.3)	84.12 (2.0)	83.12 (1.6)	82.90 (1.3)	82.83 (0.6)	83.55 (1.5)	81.97 (1.2)	82.34 (1.8)	0.068	84.4 ^a^ (1.6)	83.0 ^b^ (1.4)	83.2 ^b^ (1.1)	82.2 ^b^ (1.5)	83.1 (1.5)	83.2 (1.5)	0.008	0.346	0.731
Dressing (%)	69.62 (3.2)	72.0 (1.9)	72.68 (2.5)	70.73 (3.2)	70.12 (2.2)	69.45 (5.6)	72.39 (1.9)	69.37 (2.0)	0.241	70.8 (2.4)	71.7 (2.9)	69.8 (4.1)	70.9 (2.5)	71.2 (3.2)	70.4 (3.1)	0.635	0.274	0.073
Heart (%)	0.43 (0.03)	0.39 (0.04)	0.42 (0.05)	0.43 (0.04)	0.42 (0.07)	0.42 (0.04)	0.43 (0.08)	0.43 (0.05)	0.911	0.42 (0.04)	0.42 (0.05)	0.42 (0.05)	0.43 (0.06)	0.43 (0.05)	0.42 (0.05)	0.818	0.331	0.612
Liver (%)	2.23 (0.4)	2.01 (0.2)	2.19 (0.2)	2.07 (0.2)	2.26 (0.3)	2.04 (0.3)	2.18 (0.2)	2.16 (0.2)	0.228	2.12 (0.3)	2.13 (0.2)	2.15 (0.3)	2.17 (0.2)	2.22 (0.3)	2.07 (0.2)	0.617	0.038	0.275
Gizzard (%)	1.16 (0.1)	1.17 (0.1)	1.15 (0.1)	1.13 (0.1)	1.18 (0.09)	1.43 (0.2)	1.13 (0.1)	1.21 (0.2)	0.492	1.17 (0.1)	1.14 (0.1)	1.30 (0.2)	1.17 (0.2)	1.16 (0.2)	1.24 (0.2)	0.122	0.141	0.386
Giblets (%)	3.83 (0.5)	3.58 (0.3)	3.76 (0.4)	3.63 (0.3)	3.86 (0.4)	3.88 (0.4)	3.74 (0.3)	3.80 (0.3)	0.781	3.71 (0.4)	3.70 (0.3)	3.87 (0.4)	3.78 (0.2)	3.80 (0.3)	3.73 (0.3)	0.075	0.546	0.476
Breast (%)	19.60 (2.1)	20.80 (1.3)	21.27 (2.3)	20.39 (1.8)	20.27 (1.9)	20.08 (2.3)	20.93 (1.6)	19.17 (1.7)	0.590	20.2 (1.8)	20.8 (1.9)	20.2 (2.0)	20.1 (1.8)	20.5 (1.8)	20.1 (1.8)	0.925	0.475	0.230
Thigh (%)	8.84 (0.6)	8.95 (0.5)	8.69 (0.5)	8.74 (1.0)	8.83 (1.0)	9.32 (0.7)	8.90 (0.9)	8.52 (0.7)	0.794	8.90 (0.5)	8.71 (0.7)	9.07 (0.9)	8.71 (0.7)	8.82 (0.7)	88.88 (0.7)	0.495	0.710	0.750
Shank (%)	6.37 (0.4)	6.54 (0.3)	6.14 (0.3)	6.46 (0.3)	6.46 (0.6)	6.41 (0.3)	6.47 (0.2)	6.24 (0.2)	0.510	6.46 (0.3)	6.30 (0.3)	6.44 (0.4)	6.36 (0.2)	6.36 (0.3)	6.42 (0.3)	0.042	0.681	0.004
Abdominal fat (%)	0.96 (0.3)	0.80 (0.2)	0.59 (0.2)	0.67 (0.3)	0.64 (0.2)	0.74 (0.5)	0.77 (0.3)	0.62 (0.2)	0.423	0.88 (0.3)	0.64 (0.2)	0.69 (0.4)	0.70 (0.2)	0.75 (0.3)	0.71 (0.3)	0.323	0.637	0.745

CL: control diet low dosage; CH: control diet high dosage; AL: 3% inclusion level of apple pomace; AH: 6% inclusion level of apple pomace; BL: 3% inclusion level of blackcurrant pomace; BH: 6% inclusion level of blackcurrant pomace; SL: 3% inclusion level of strawberry pomace; SH: 6% inclusion level of strawberry pomace; L&F: lean tissue and fluid content; ACL: integral antioxidant capacity of lipophilic substances; ACW: integral antioxidant capacity of hydrophilic substances; FRAP: ferric reducing ability; SEM, standard error of the mean. * *p*-value of one-way ANOVA or Kruskal–Wallis tests. ^#^ *p*-value of robust two-way ANOVA. Means with different superscript letters (a and b) within the same column per fixed effect (i.e., pomace and dosage) differ significantly (*p* < 0.05).

**Table 4:** Serum antioxidant parameters, serum lipids and minerals and renal and liver function.

	Groups								*p*-Value *	Pomace (P)				Dosage (D)		*p*-Value P ^#^	*p*-ValueD ^#^	*p*-ValueP × D ^#^
	CH	CL	AH	AL	BH	BL	SH	SL		C	A	B	S	L	H			
FRAP ^1^	0.13 (0.01)	0.13 (0.01)	0.13 (0.02)	0.14 (0.03)	0.09 (0.01)	0.13 (0.04)	0.11 (0.02)	0.15 (0.07)	0.127	0.13 (0.01)	0.14 (0.02)	0.11 (0.03)	0.13 (0.05)	0.11 (0.02)	0.14 (0.03)	0.561	0.157	0.518
ACW ^1^	16.69 (2.2)	16.56 (2.7)	19.08 (1.4)	25.63 (4.7)	12.62 (1.8)	14.32^a^ (5.7)	12.05 (1.5)	21.96 (5.9)	<0.001	16.6 ^a^ (2.4)	22.4 ^b^ (4.7)	13.5 ^a^ (4.1)	17.0 ^a^ (6.6)	15.1 ^a^ (5.3)	19.6 ^b^ (5.9)	0.008	0.004	0.089
ACL ^1^	5.4 (1.1)	5.6 (1.0)	3.4 (0.7)	6.4 (1.9)	4.1 (0.9)	3.7 (0.7)	3.6 (0.9)	3.8 (1.4)	<0.001	5.47 ^a^ (1.0)	4.89 ^a^ (2.1)	3.93 ^b^ (0.8)	3.75 ^b^ (1.1)	4.13 ^a^ (1.5)	4.89 ^b^ (1.5)	0.003	0.057	0.027
AIP ^2^	−0.29 (0.1)	−0.29 (0.1)	−0.45 (0.1)	−0.53 (0.07)	−0.55 (0.09)	−0.56 (0.1)	−0.48 (0.2)	−0.56 (0.1)	<0.001	−0.29 ^a^ (0.1)	−0.49 ^b^ (0.1)	−0.56 ^b^ (0.09)	−0.52 ^b^ (0.1)	−0.45 (0.1)	−0.48 (0.1)	0.001	0.441	0.382
Trigly ^3^	0.89 (0.22)	0.92 (0.17)	0.67 (0.18)	0.50 (0.07)	0.52 (0.12)	0.56 (0.15)	0.64 (0.25)	0.52 (0.14)	<0.001	0.91^a^ (0.2)	0.58 ^b^ (0.1)	0.54 ^b^ (0.1)	0.58 ^b^ (0.2)	0.68 (0.2)	0.63 (0.2)	0.001	0.500	0.095
HDL ^3^	54.6 (3.9)	55.8 (3.5)	62.4 (4.2)	59.6 (4.5)	60.5 (2.2)	61.8 (10.2)	59.9 (11.1)	60.6 (2.7)	0.078	1.75 (0.2)	1.77 (0.1)	1.91 (0.3)	1.86 (0.2)	1.82 (0.2)	1.83 (0.2)	0.421	0.739	0.092
Non-HDL ^3^	1.43 (0.2)	1.42 (0.2)	1.13 (0.2)	1.15 (0.2)	1.19 (0.1)	1.22 (0.3)	1.25(1.1)	1.20(1.1)	0.108	1.43 (0.2)	1.14 (0.2)	1.21 (0.2)	1.23 (0.1)	1.25 (0.2)	1.25 (0.2)	0.115	0.864	0.816
AST ^4^	225.0 (18.3)	263.5 (45.4)	214.1 (30.5)	243.0 (22.2)	248.0 (34.8)	210.2 (12.5)	244.4 (34.7)	250.6 (39.7)	0.061	244 ^a^ (38.6)	229 ^b^ (29.6)	229 ^b^ (31.8)	247 ^a^ (35.7)	233 (33.4)	242 (35.6)	0.022	0.138	0.003
ALT ^4^	7.78 (1.7)	9.30 (2.4)	8.98 (3.4)	6.21 (1.4)	6.02 (1.1)	8.28 (3.1)	9.47 (2.8)	7.57 (1.2)	0.080	8.54 (2.1)	7.60 (2.9)	7.15 (2.5)	8.52 (2.3)	8.06 (2.6)	7.84 (2.5)	0.548	0.749	0.143
ALT ^4^	3837 (1116)	3695 (1574)	4508 (1472)	3547 (1172)	2653 (859.9)	4008 (1423)	3199 (1313)	4055 (1432)	0.368	3766 (1302)	4027 (1363)	3331 (1325)	3627 (1384)	3549 (1319)	3826 (1363)	0.809	0.628	0.238
Uric acid ^5^	335.0 (43.7)	377.7 (98.6)	310.2 (68.2)	371.7 (79.6)	344.0 (85.9)	327.7 (64.1)	384.0 (49.1)	401.2 (58.2)	0.321	356 (76.1)	341 (77.6)	336 (72.8)	393 (52.1)	343 (71.8)	370 (74.7)	0.305	0.279	0.684
Crea ^5^	5.83 (2.5)	6.43 (3.2)	5.08 (3.8)	5.73 (4.2)	3.70 (1.6)	2.71 (0.5)	2.17 (1.1)	3.30 (1.2)	0.050	6.13 ^a^ (2.8)	5.41 ^ab^ (3.8)	3.21 ^b^ (1.2)	2.73 ^b^ (1.2)	4.20 (2.9)	4.55 (2.8)	0.021	0.683	0.475
Ca ^6^	1.69 (0.1)	1.79 (0.2)	1.78 (0.2)	1.88 (0.3)	1.81 (0.2)	1.85 (0.1)	1.96 (0.3)	1.84 (0.2)	0.641	1.74 (0.2)	1.83 (0.3)	1.83 (0.2)	1.90 (0.2)	1.81 (0.2)	1.84 (0.2)	0.397	0.800	0.818
P ^6^	1.64 (0.2)	1.55 (0.2)	1.42 (0.2)	1.41 (0.2)	1.39 (0.2)	1.35 (0.1)	1.34 (0.1)	1.37 (0.07)	0.076	1.60 (0.2)	1.42 (0.2)	1.37 (0.2)	1.36 (0.1)	1.45 (0.2)	1.42 (0.2)	0.029	0.667	0.649
Mg ^7^	0.77 (0.02)	0.75 (0.04)	0.75 (0.06)	0.72 (0.04)	0.76 (0.07)	0.73 (0.04)	0.79 (0.02)	0.79 (0.08)	0.175	0.76 (0.03)	0.74 (0.04)	0.74 (0.06)	0.79 (0.05)	0.77 (0.04)	0.75 (0.05)	0.430	0.067	0.859

CL: control diet with 3% of cellulose; CH: control diet with 6% of cellulose; AL: 3% inclusion level of apple pomace; AH: 6% inclusion level of apple pomace; BL: 3% inclusion level of blackcurrant pomace; BH: 6% inclusion level of blackcurrant pomace; SL: 3% inclusion level of strawberry pomace; SH: 6% inclusion level of strawberry pomace. ACL: integral antioxidant capacity of lipophilic substances; ACW: integral antioxidant capacity of hydrophilic substances; AIP: atherogenic index of plasma; ALT: alanine transaminase; AST: aspartate transaminase; ALP: alkaline phosphatase; Crea: creatinine; FRAP: ferric reducing ability; HDL: high-density lipoprotein; Trigly: triglycerides. ^1^ Milligram of Trolox per millilitre; ^2^ lg (triglycerides/HDL); ^3^ mmol/L; ^4^ U/L; ^5^ µmol/L; ^6^ mg/dL; ^7^ ug/dL. * *p*-value of one-way ANOVA or Kruskal–Wallis tests. ^#^ *p*-value of robust two-way ANOVA. Means with different superscript letters (a and b) within the same column per fixed effect (i.e., pomace and dosage) differ significantly (*p* < 0.05).

**Table 5:** Breast and liver antioxidant parameters.

	Groups								*p*-Value *	Pomace (P)				Dosage (D)		*p*-Value P ^#^	*p*-Value D ^#^	*p*-Value P × D ^#^
	CH	CL	AH	AL	BH	BL	SH	SL		C	A	B	S	L	H			
**Breast**																		
FRAP ^1^	0.22 (0.01)	0.24 (0.04)	0.24	0.26	0.25	0.27	0.23	0.26	0.071	0.23 ^a^ (0.03)	0.25 ^bc^ (0.03)	0.26 ^bc^ (0.03)	0.25 ^ac^ (0.03)	0.24 ^a^ (0.03)	0.26 ^b^ (0.03)	0.009	0.017	0.595
ACW ^1^	0.14 (0.008)	0.15 (0.01)	0.13 (0.008)	0.13 (0.02)	0.13 (0.02)	0.18 (0.10)	0.15 (0.01)	0.16 (0.02)	0.019	0.15 ^a^ (0.01)	0.14 ^b^ (0.01)	0.16 ^b^ (0.07)	0.16 ^b^ (0.01)	0.14(0.04)	0.16(0.04)	0.007	0.101	0.812
ACL^1^	0.49(0.01)	0.53(0.03)	0.52(0.02)	0.51 (0.02)	0.51 (0.04)	0.60 (0.2)	0.55 (0.3)	0.57 (0.06)	0.029	0.51 ^a^ (0.02)	0.51 ^a^ (0.03)	0.56 ^a^ (0.14)	0.57 ^b^ (0.04)	0.52 (0.08)	0.55 (0.08)	0.008	0.343	0.197
**Liver**																		
FRAP ^1^	3.25 (0.4)	3.42 (0.2)	3.41 (0.3)	3.54 (0.3)	3.23 (0.1)	3.43 (0.2)	3.43 (0.4)	3.47 (0.1)	0.557	3.33 (0.03)	3.48 (0.3)	3.33 (0.2)	3.45 (0.32)	3.33 (0.3)	3.47 (0.3)	0.186	0.080	0.801
ACW ^1^	1.76 (0.09)	1.86 (0.1)	1.89 (0.2)	2.07 (0.2)	1.96 (0.1)	2.02 (0.1)	2.04 (0.3)	2.03 (0.05)	0.070	1.81 (0.1)	1.98 (0.2)	1.99 (0.1)	2.03 (0.2)	1.91 (0.2)	1.99 (0.2)	0.055	0.276	0.987
ACL ^1^	3.79 (0.3)	3.89 (0.3)	3.68 (0.2)	4.54 (0.4)	4.46 (0.1)	4.55 (0.2)	4.37 (0.3)	4.20 (0.3)	<0.001	3.84 ^a^ (0.2)	4.11 ^a^ (0.5)	4.51 ^b^ (0.1)	4.29 ^b^ (0.3)	4.08 ^a^ (0.4)	4.30 ^b^ (0.4)	0.001	0.036	0.043
TBARS ^2^	3.65 (1.3)	3.73 (0.8)	3.60 (1.2)	3.75 (0.9)	3.13 (0.8)	4.19 (1.3)	3.75 (1.2)	2.8 (0.7)	0.479	3.69 (1.0)	3.68 (1.0)	3.66 (1.2)	3.29 (1.04)	3.53 (1.05)	3.63 (1.0)	0.779	0.618	0.333
GSH ^3^	192.4 (21.2)	188.1 (11.6)	189.5 (26.5)	191.5 (13.3)	218.1 (44.4)	214.2 (44.7)	200.0 (18.9)	181.0 (14.4)	0.247	190 (17.3)	191 (19.9)	215 (42.9)	191 (18.5)	200 (28.9)	194 (29.1)	0.576	0.565	0.757
GSSG ^3^	2.38 (0.5)	2.24 (0.5)	1.59 (0.33)	1.99 (0.3)	2.15 (0.5)	2.06 (0.7)	2.31 (0.4)	1.39 (0.3)	0.003	2.31 ^a^ (0.5)	1.79 ^b^ (0.4)	2.11 ^a^ (0.6)	1.85 ^a^ (0.6)	2.11 (0.5)	1.92 (0.5)	0.052	0.129	0.006
GSH/GSSG	83.02 (14.0)	86.62 (16.9)	120.5 (11.0)	97.21 (9.2)	104.0 (18.8)	106.8 (16.7)	88.03 (10.9)	133.3 (22.7)	<0.001	84.8 ^a^ (14.9)	109.0 ^b^ (15.6)	105.0 ^b^ (17.0)	111.0 ^b^ (29.1)	98.9 (18.2)	106 (21.6)	0.017	0.235	0.001

CL: control diet with 3% of cellulose; CH: control diet with 6% of cellulose; AL: 3% inclusion level of apple pomace; AH: 6% inclusion level of apple pomace; BL: 3% inclusion level of blackcurrant pomace; BH: 6% inclusion level of blackcurrant pomace; SL: 3% inclusion level of strawberry pomace; SH: 6% inclusion level of strawberry pomace. SEM: standard error of the mean; ACL: integral antioxidant capacity of lipophilic substances; ACW: integral antioxidant capacity of hydrophilic substances; FRAP: ferric reducing ability; GSH: reduced glutathione; GSSG: oxidized glutathione. ^1^ Milligram of Trolox per gram dry matter; ^2^ µmol/kg; ^3^ µmol/g. * *p*-value of one-way ANOVA or Kruskal–Wallis tests. ^#^ *p*-value of robust two-way ANOVA. Means with different superscript letters (a and b) within the same column per fixed effect (i.e., pomace and dosage) differ significantly (*p* < 0.05).

**Table 6:** Histopathological scores of spleen, liver, thymus and bursa of Fabricius.

	Groups								*p*-Value *	Pomace (P)				Dosage (D)		*p*-Value P ^#^	*p*-Value D ^#^	*p*-Value P ×D ^#^
	CL	CH	AL	AH	BL	BH	SL	SH		C	A	B	S	L	H			
**Organ**																		
Spleen	0.17 (0.4)	0.17 (0.4)	0.17 (0.4)	0.0 (0.0)	0.17 (0.4)	0.25 (0.4)	0.50 (0.5)	0.17 (0.4)	0.652	0.17 (0.38)	0.08 (0.3)	0.21 (0.4)	0.33 (0.5)	0.14 (0.4)	0.25 (0.4)	0.503	0.378	0.602
Liver	0.83 (0.6)	1.50 (1.22)	1.58 (1.1)	1.25 (1.1)	1.00 (0.6)	1.33 (0.9)	1.33 (0.9)	0.58 (0.7)	0.825	1.17 (1.0)	1.41 (1.0)	1.17 (0.8)	0.96 (0.8)	1.17 (0.9)	1.18 (0.9)	0.721	0.784	0.306
Thymus	Absence of alterations																	
Bursa of Fabricius	1.41 (0.5)	0.83 (0.9)	0.83 (0.7)	1.08 (0.9)	0.66 (0.7)	1.00 (0.5)	0.58 (0.7)	0.58 (0.7)	0.532	1.12 (0.7)	0.96 (0.8)	0.83 (0.6)	0.58 (0.6)	0.87 (0.7)	0.87 (0.7)	0.564	0.833	0.601

* *p*-value of one-way ANOVA or Kruskal–Wallis tests. ^#^ *p*-value of robust two-way ANOVA.
